# The Impact of Social Media Marketing Activities on Consumer Inspiration, Food Pleasure, and Behavioral Intentions: Evidence from Dubai Chocolate

**DOI:** 10.3390/foods15061097

**Published:** 2026-03-20

**Authors:** Handan Hamarat, Sinan Çavuşoğlu, Murat Göral, Yusuf Gökçe, Ahmet Uslu, Aziz Bükey

**Affiliations:** 1 Hotel, Restaurant and Catering Services Department, Bingöl University, Bingöl 12000, Türkiye; hhamarat@bingol.edu.tr (H.H.); mgoral@bingol.edu.tr (M.G.); ygokce@bingol.edu.tr (Y.G.); abukey@bingol.edu.tr (A.B.); 2 Finance, Banking and Insurance Department, Bingöl University, Bingöl 12000, Türkiye; 3 Office Services and Secretarial Department, Bingöl University, Bingöl 12000, Türkiye; ahmetuslu@bingol.edu.tr

**Keywords:** social media marketing activities, consumer inspiration, food pleasure, behavioral intentions

## Abstract

This study investigates how innovative social media marketing activities influence consumer inspiration, food pleasure, and behavioral intentions in the context of hedonic food consumption and digital marketing innovation. Data collected from 425 consumers who had tried Dubai chocolate products in Türkiye were analyzed using the partial least squares structural equation modeling (PLS-SEM) method with SmartPLS 4 software. The results indicate that personalization, trendiness, and advertisement dimensions significantly enhance consumer inspiration, whereas entertainment and interaction dimensions show no significant effects. Consumer inspiration positively influences repurchase intention, recommendation intention, willingness to pay more, and food pleasure. Furthermore, food pleasure exerts a significant positive effect on recommendations and willingness to pay more but not on repurchase intention. Mediation analysis revealed that food pleasure partially mediates the relationships between consumer inspiration, recommendation intention, and willingness to pay more, whereas no mediating effect was found for repurchase intention. These findings contribute to innovation and knowledge literature by demonstrating how digital marketing activities foster emotional engagement, enhance consumer experiences, and promote sustainable behavioral intentions in the hedonic food sector.

## 1. Introduction

Over the past decade, digital transformation has reshaped how brands communicate with consumers through social media. Beyond information dissemination and awareness, social media increasingly functions as a strategic space where brands can evoke emotions, create meaning, and stimulate experience-oriented engagement [1,2]. This shift is especially relevant in hedonic consumption contexts, where consumers evaluate offerings not only for functional benefits but also for their emotional and sensory value.

Social media marketing activities (SMMAs)—including entertainment, interaction, personalization, trendiness, and advertising—can influence consumers’ cognitive and affective responses and shape brand-related experiences [3]. Prior research has linked SMMAs to outcomes such as engagement, trust, loyalty, and brand image [4,5]. However, their role in shaping consumer inspiration, defined as a cognitive-emotional state triggered by external cues that motivate new ideas or actions, has received comparatively less attention [6,7]. More importantly, existing studies often provide limited clarity on how inspiration translates into downstream consumption-related outcomes in hedonic settings.

Against this backdrop, this study examines the effects of SMMAs on consumer inspiration and clarifies the mechanism through which inspiration relates to food pleasure and behavioral intentions. We focus on Dubai Chocolate as a hedonic food context characterized by strong sensory associations and intense social-media visibility [8,9]. As a visually marketable and trend-driven product, its diffusion is closely linked to short-form videos, visually rich promotional content, and user-generated posts, making it a suitable setting to observe how SMMAs may trigger inspiration and shape pleasure-based outcomes, such as word-of-mouth (WOM) and willingness to pay more (WPM). In addition, its positioning as a premium/indulgent experience makes food pleasure theoretically central for explaining how inspiration relates to subsequent intentions.

This study contributes to the literature in three ways. First, it extends SMMA research by focusing on consumer inspiration and positioning food pleasure as an affective pathway connecting inspiration to behavioral outcomes in a hedonic food context. Second, grounded in hedonic consumption theory [10] and the stimulus–organism–response (SOR) paradigm [11], the model integrates social media stimuli, organismic states (inspiration and pleasure), and responses (behavioral intentions) into a coherent mechanism. Rather than proposing an entirely new theory, this study synthesizes these established perspectives to clarify how inspiration and food pleasure jointly explain behavioral outcomes in a social-media-driven hedonic food setting. Third, by providing evidence from Türkiye, an emotionally engaged and highly active social-media market [12,13], the study offers context-specific insights for food brands seeking to design inspiration-triggering digital strategies.

## 2. Conceptual Framework

### 2.1. SMMAs

Social media has become an integral component of contemporary marketing practice and a key channel for reaching and engaging consumers [14]. As a subdomain of digital marketing, social media marketing leverages platform-specific features to support corporate objectives through stakeholder value creation [15]. In this context, SMMAs are widely viewed as strategic practices that can shape consumer experience, satisfaction, and behavioral intentions [3,16,17]. Prior research has shown that SMMAs strengthen consumer–brand engagement and influence brand image, trust, loyalty, and purchase-related intentions [18,19,20].

Although SMMAs have been conceptualized using different frameworks, a frequently adopted classification comprises five dimensions: entertainment, interaction, customization, trendiness, and advertisement [3,21,22]. These dimensions are particularly relevant in hedonic consumption contexts, where marketing stimuli can trigger emotional responses and meaning-making processes that underpin inspiration and subsequent behavioral outcomes. Entertainment reflects the extent to which social media content is enjoyable and emotionally appealing, which may generate favorable affect toward the brand [23]. Interaction captures two-way communication and consumer-to-consumer exchange on social platforms, enabling users to share opinions and experiences [22]. Such interactions can reinforce loyalty and, in food-related contexts, shape perceptions of products and consumption experiences [19].

Trendiness refers to the provision of up-to-date and fashionable information on social media. For food brands, posts featuring new flavors, limited editions, or creative gastronomic experiences may stimulate curiosity and foster inspiration [4]. Advertisement refers to promotional campaigns and paid/owned marketing initiatives delivered through social media. In hedonic product categories, advertising often emphasizes not only functional attributes but also the product’s potential to provide sensory pleasure and emotional enjoyment [2].

### 2.2. Consumer Inspiration

The concept of inspiration originates in social psychology and is commonly described as a dual process comprising two phases: “inspired-by” and “inspired-to” [6,24]. The inspired-by phase refers to the reception and recognition of novel ideas triggered by external stimuli, whereas the inspired-to phase reflects the motivational impulse to act on those ideas [25,26]. Accordingly, inspiration is a transient yet influential psychological state characterized by the co-occurrence of cognitive awareness and motivational activation [27]. In marketing, consumer inspiration is defined as a motivational state in which consumers perceive new consumption possibilities through marketing stimuli and feel compelled to pursue them [7]. As such, consumer inspiration can facilitate discovery, strengthen engagement, and increase consumption-related intentions [28,29]. Prior research suggests that inspiration may be elicited by innovative products, creative advertising, personalized recommendations, and experiential campaigns [7,30]. The literature also identifies three core features of consumer inspiration: transcendence (envisioning improved possibilities), evocation (being triggered by external stimuli rather than self-generated), and motivation (energizing action) [24,31]. Together, these features provide a basis for expecting that inspiration is associated with hedonic experiences and subsequent behavioral intentions.

### 2.3. Behavioral Intentions

Behavioral intention refers to an individual’s stated likelihood or plan to purchase, use, or recommend a product or service in the future [32]. It is widely considered a proximal predictor of subsequent behavior and is shaped by consumers’ perceptions, attitudes, and prior experiences [33,34]. In food and gastronomy contexts—particularly within hedonic consumption—behavioral intentions are closely linked to consumption experiences, as enjoyment and positive affect can increase consumers’ propensity to repurchase, recommend, and accept higher prices [35,36,37]. Accordingly, behavioral intention provides a useful link between consumer experience and value-related market outcomes.

In the present study, behavioral intentions are examined through three dimensions. Repurchase intention reflects the tendency to choose the same product or brand again and is associated with perceived value and satisfaction [38,39]. WOM intention captures consumers’ willingness to share positive information about a product or brand and is often reinforced by satisfaction and trust, offering firms an important non-paid communication channel, especially in social media environments where recommendations diffuse rapidly [40,41,42]. WPM refers to consumers’ readiness to pay a premium compared to available alternatives and is commonly related to perceived value, quality perceptions, and brand attachment [43,44]. A higher willingness to pay can support value-oriented strategies and strengthen competitive advantage [36].

### 2.4. Food Pleasure

Food is not merely a physiological necessity but a multifaceted experience encompassing sensory, emotional, and social dimensions. In the literature, pleasure is recognized as a fundamental psychological construct associated with positive emotions [45] and is emphasized as a core source of hedonic satisfaction in food consumption [46]. Food pleasure arises not only from multisensory attributes such as taste, aroma, texture, and visual appeal but also from the social context and cultural meanings surrounding eating [8,9].

Eating experiences provide more than satiation; they deliver comfort, enjoyment, and emotional fulfillment [47]. Consequently, food pleasure has become an increasingly prominent topic in consumer behavior research. Within hedonic consumption, eating may intertwine with nostalgic associations, social rituals, and aesthetic presentation, thereby eliciting more intense pleasure responses [48]. With the proliferation of social media, food pleasure has also become more visible and performative through visual sharing, food photography, and online interactions, which can reinforce consumers’ emotional bonds with food [49,50]. Recent studies further suggest that food-related pleasure is not exclusively rooted in sensory experiences but is also shaped by social dynamics. Shared dining, celebrations, and gatherings with family or friends are identified as contextual elements that enhance enjoyment [51,52]. Accordingly, food pleasure is often conceptualized as a multidimensional construct that encompasses both individual sensory gratifications (for example, taste, smell, texture, and visual attractiveness) and socially embedded experiences [53,54]. This perspective positions food not only as nourishment but also as a vehicle of emotional connection, cultural expression, and meaning making.

To clarify the conceptual boundaries of the construct, food pleasure (pleasure of eating) is treated as an overall hedonic evaluation of the eating experience, that is, the extent to which consuming a given food elicits enjoyment and positive affect [45,47]. This evaluation is not limited to immediate sensory gratification because sensory inputs are interpreted through affective and symbolic processes during consumption [46,48]. Accordingly, sensory pleasure (taste, aroma, texture, and visual appeal) represents the most proximal source of enjoyment [46,54], whereas elements such as nostalgia and aesthetic appreciation are conceptualized as affective meaning components that can intensify or shape felt pleasure by linking the consumption episode to personally meaningful memories and emotions [48]. Likewise, the social context (e.g., sharing food, rituals, and celebrations) is considered part of food pleasure because it can amplify positive affect during consumption and influence how pleasurable the episode is perceived [51,52]. Consistent with this perspective, we conceptualize food pleasure as a multidimensional experiential construct that captures (i) sensory gratification, (ii) affective meaning (e.g., nostalgic feelings), and (iii) social enjoyment, which together reflect the overall pleasurable quality of eating in hedonic consumption settings [53,54].

## 3. Theoretical Framework

### 3.1. The Effect of SMMAs on Consumer Inspiration

SMMAs constitute a dynamic communication mechanism through which brands engage with consumers, foster emotional connections, and deliver content with inspirational potential [5]. Social media has been described as Internet-based applications that enable the creation and exchange of user-generated content [1]. Such environments allow brands to not only disseminate information but to also design creative and emotionally resonant content that captures attention and stimulates consumer responses [55]. In line with this view, Böttger’s Customer Inspiration Cycle Model emphasizes that inspiration can develop from initial awareness to emotional activation and ultimately to behavioral action, suggesting that visually rich and engaging digital content may support inspiration-related processes across these stages [56].

Consumer inspiration is commonly treated as a two-dimensional construct comprising cognitive activation (“inspired-by”) and motivational activation (“inspired-to”) [6]. Within social media contexts, brand-generated content that is entertaining, interactive, customized, trend-focused, and creatively presented can facilitate engagement and meaning-making, thereby increasing the likelihood of inspirational experiences [57,58,59]. Visually rich formats (for example, images, short videos, and animations) and storytelling can communicate cues at both the cognitive and affective levels and may stimulate imagination and novel thinking [25,60]. Consistent with this perspective, digital media can serve as a key trigger of consumer inspiration by combining creativity with emotional connection [30].

Social influence and curiosity may further reinforce inspiration in online communities. Users observe others’ posts, follow emerging trends, and discover alternative product uses, which can increase cognitive stimulation and motivate novelty-seeking [61,62]. Prior empirical research supports these associations. For example, Ghafourzay and Parıltı [5] reported positive effects of SMMA dimensions on consumer inspiration, and Sharma et al. [55] similarly found that SMMAs positively influence consumer inspiration. Sheng et al. [63] also reported that advertising- and interaction-related activities contribute to consumer inspiration.

Accordingly, the following hypotheses were proposed regarding the effects of SMMAs on consumer inspiration:

**H1a.**  *Entertainment positively affects consumer inspiration*.

**H1b.**  *Interaction positively affects consumer inspiration*.

**H1c.**  *Customization positively affects consumer inspiration*.

**H1d.**  *Trendiness positively affects consumer inspiration*.

**H1e.**  *Advertisement positively affects consumer inspiration*.

### 3.2. The Effect of Consumer Inspiration and Behavioral Intentions

Consumer inspiration is a transient psychological state characterized by both cognitive awareness and motivational activation triggered by external stimuli (e.g., brand messages or social media content) [7]. It consists of two interrelated components: cognitive arousal (recognizing a new possibility) and motivational activation (the urge to act on that possibility) [25]. Accordingly, inspiration operates as a psychological mechanism that translates marketing stimuli into goal-directed consumer responses.

The customer inspiration Model conceptualizes this process through the inspired-by and inspired-to phases [7]. The inspired-by component reflects the reception of brand-related stimuli and the associated affective/cognitive activation, whereas inspired-to captures the motivational impulse that drives consumers toward action. Thus, inspiration implies more than favorable attitudes; it can produce tangible behavioral tendencies.

Within this framework, consumer inspiration is expected to strengthen behavioral intentions, such as repurchase intention, WOM intention, and WPM. Inspired consumers may perceive product value and differentiation as more meaningful, which can increase repurchase likelihood [7]. Inspiration may also encourage sharing and advocacy by motivating consumers to communicate their experiences to others [64,65,66,67]. In addition, by enhancing perceived value and emotional significance, inspiration can increase consumers’ willingness to pay a premium [6,36]. Prior empirical studies support these links, showing that inspiration stimulated by social media marketing is positively associated with behavioral intentions [5,68,69].

Accordingly, the following hypotheses were proposed:

**H2a.**  *Consumer inspiration positively affects repurchase intention*.

**H2b.**  *Consumer inspiration positively affects WOM intention*.

**H2c.**  *Consumer inspiration positively affects WPM*.

### 3.3. The Effects Among Consumer Inspiration, Food Pleasure, and Behavioral Intentions

Consumer inspiration is a transient motivational state that comprises cognitive awareness (“inspired-by”) and motivational activation (“inspired-to”) [6]. It emerges when external marketing stimuli, such as social media content, generate novel consumption ideas and simultaneously evoke an intrinsic drive to pursue those ideas [7].

The SOR paradigm and pleasure–arousal–dominance (PAD) framework provide a useful basis for explaining how external stimuli shape internal organismic states and, in turn, behavioral outcomes. In this study, SMMAs represent the stimuli (S), consumer inspiration and food pleasure capture the organismic states (O), and behavioral intentions (repurchase, WOM, and WPM) represent the responses (R) [13,70]. These frameworks are particularly relevant for understanding the emotional (pleasure-related) and behavioral (intention-driven) consequences of inspiration in hedonic food consumption.

In hedonic consumption contexts, inspiration can stimulate curiosity and exploration related to new flavors, presentation styles, and consumption rituals. External cues, such as innovative food presentation, visually appetizing imagery, and narrative storytelling, can heighten sensory expectations and intensify perceived food pleasure [8,53,71]. For instance, inspired consumers may evaluate novel flavor combinations or aesthetic packaging more favorably, which can enhance the pleasure experienced during consumption. Accordingly, we propose:

**H3.**  *Consumer inspiration positively affects food pleasure*.

Food pleasure represents a central hedonic component of consumer experience and is often associated with behavioral intentions. Prior research in hospitality and consumer behavior reports positive links between pleasurable consumption experiences and repurchase intention, recommendation/WOM, and willingness to pay a premium [36,72,73,74,75,76]. Pleasurable experiences can strengthen repurchase tendency by reinforcing positive memories and confirming expectations [36,77]. They can also stimulate social sharing and positive WOM, as satisfaction and enjoyment motivate consumers to recommend products to others [73,74]. Moreover, higher emotional value may increase perceived value and reduce price sensitivity, thereby raising WPM [36,44]. Based on these insights, we propose:

**H4a.**  *Food pleasure positively affects repurchase intention*.

**H4b.**  *Food pleasure positively affects WOM intention*.

**H4c.**  *Food pleasure positively affects WPM*.

Finally, consumer inspiration may influence behavioral intentions directly and indirectly through food pleasure. Consistent with the SOR framework and affect-based models of consumer behavior, inspiration can be translated into intentions via an emotional pathway (inspiration → food pleasure → intentions). Previous research has supported the mediating role of pleasure in shaping usage-related and behavioral outcomes [78]. Therefore, we propose:

**H5.**  *Food pleasure mediates the relationship between consumer inspiration and repurchase intention*.

**H6.**  *Food pleasure mediates the relationship between consumer inspiration and WOM intention*.

**H7.**  *Food pleasure mediates the relationship between consumer inspiration and WPM*.

## 4. Method

**Figure 1:**
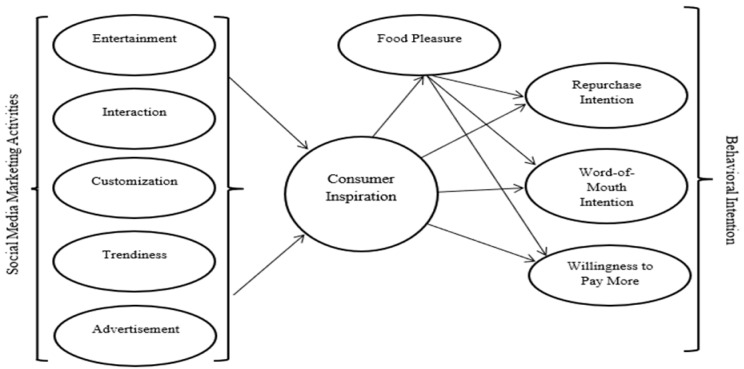
Research Model.

This study examines how SMMAs shape consumer inspiration and its downstream associations with food pleasure and behavioral intentions in a hedonic food consumption context (See Figure 1). A quantitative survey-based research design was employed to test the proposed conceptual model. The target population comprised individuals residing in Türkiye who actively used social media and consumed Dubai chocolate at least once. Participants were recruited via social media channels using a non-probability convenience sampling approach. The eligibility criteria required respondents to (1) have an active social media account and (2) report prior experience with the focal product, ensuring familiarity with the consumption context. This approach enabled access to the consumer group most relevant to the research objective (such as socially connected users exposed to social media-driven food trends), while we acknowledge the associated generalizability limitations in the manuscript.

The measurement items for the five SMMA dimensions (entertainment, interaction, customization, trendiness, and advertising) were adapted from Bilgin [3]. Consumer inspiration was measured using items adapted from Böttger et al. [7], and food pleasure was measured using the scale developed by Cao et al. [71]. Behavioral intentions (repurchase intention, WPM, and WOM intention) were adapted from Bushara et al. [41]. All items were assessed using a five-point Likert scale ranging from 1 (“strongly disagree”) to 5 (“strongly agree”). Operational definitions and measurement items are presented in Table 1.

Data were collected in the last quarter of 2025. After screening for completeness and consistency, 425 valid responses were retained for analysis. The sample size was adequate for partial least squares structural equation modeling (PLS-SEM) and provided sufficient power to estimate the proposed model and test the hypothesized relationships.

### Data Analyses

Data analysis was conducted using SmartPLS 4. The proposed model linking SMMAs to consumer inspiration and behavioral intentions, with food pleasure as a mediator, was estimated using the partial least squares approach. This technique was selected because it is well suited for estimating complex models with multiple latent constructs and mediating relationships and for evaluating both explanatory and predictive performance.

The measurement model was assessed by examining internal consistency reliability and convergent validity using Cronbach’s alpha (α), composite reliability (CR), average variance extracted (AVE), and outer loadings. Discriminant validity was evaluated using the Fornell–Larcker criterion and the heterotrait–monotrait ratio (HTMT). For the structural model, multicollinearity was assessed using inner variance inflation factor (VIF) values, while the coefficient of determination (R^2^) and effect size (f^2^) were used to evaluate explanatory power and effect sizes. The significance of the hypothesized relationships was tested using bootstrapping with 5000 resamples to obtain robust standard errors and confidence intervals for the path coefficients.

## 5. Results

The demographic characteristics of the survey respondents are first outlined, and detailed results are presented in Table 1.

A total of 425 valid responses were included in the analysis. As presented in Table 1, most participants were young adults, with 29.6% aged between 18 and 24 years and 24.0% between 25 and 34 years, indicating that the sample primarily consisted of individuals representing the digitally active generation. The gender distribution was nearly balanced, comprising 51.5% male and 48.5% female. With respect to marital status, single participants accounted for 56.7% of the sample, whereas married individuals represented 43.3%. Regarding educational attainment, most respondents held a bachelor’s degree (44.0%), followed by associate degree holders (29.6%) and those with a master’s or doctoral qualification (14.4%). In terms of perceived income, the majority described their economic situation as average (40.5%) or low (24.9%), while a smaller group reported very high-income levels (6.4%), suggesting an overall moderate socioeconomic profile. Participants reported their income level in categorical ranges based on self-perceived income status (very low to very high), which was used solely for demographic profiling. Income information was collected in broad categories (self-reported perceived level) for demographic purposes only. The survey was anonymous; no identifying information was collected, and the results are reported in aggregate form in accordance with ethical approval. Concerning social media usage, Instagram (27.3%) and TikTok (19.1%) were the most frequently used platforms, followed by YouTube (15.5%) and Facebook (14.8%). The remaining respondents reported using X (12.0%), Snapchat (8.9%), or other minor platforms (2.4%).

### 5.1. Measurement Model Assessment

**Figure 2:**
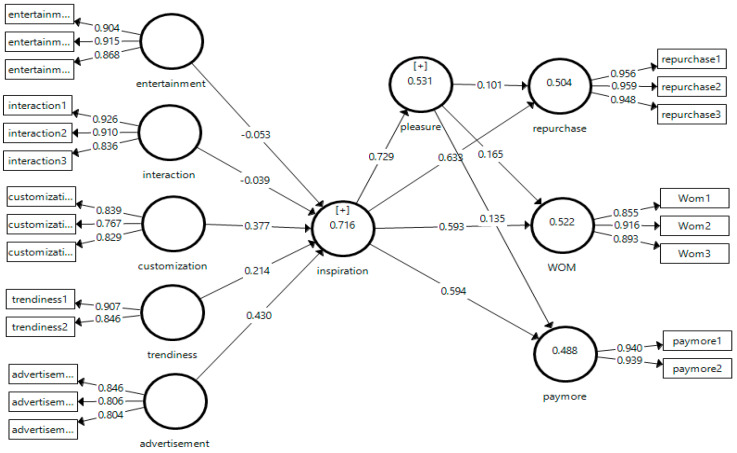
Reliability and validity.

The measurement model was assessed to evaluate the reliability and validity of the constructs, following the guidelines of Hair et al. [79,80]. Both convergent and discriminant validity were examined. In the first step, the outer loadings of all indicators were inspected, and the results indicated that most item loadings exceeded the acceptable threshold of 0.50, confirming their contribution to the corresponding latent constructs (See Figure 2) [81]. Items with slightly lower loadings were retained only if their theoretical relevance and overall construct reliability remained satisfactory.

Convergent validity was assessed using the AVE and CR indices. According to the criteria proposed by Fornell and Larcker [82], an AVE value of 0.50 or higher indicates adequate convergent validity, while CR values exceeding 0.70 demonstrate satisfactory construct reliability [83]. To further evaluate internal consistency, the α coefficients were examined, as recommended by Marmaya et al. [84] and Hair et al. [85]. In this study, all α values were above 0.70, suggesting a high level of internal reliability across the constructs. Similarly, all CR values surpassed the recommended threshold of 0.70, and AVE values exceeded 0.50, thereby confirming acceptable convergent validity for the measurement model. The overall results, presented in Table 2, indicate that the measurement model demonstrates a robust reliability structure and satisfactory model fit. Hence, the measurement framework was deemed both statistically reliable and conceptually valid, supporting its appropriateness for subsequent structural model analysis.

To evaluate discriminant validity, both the HTMT ratio and Fornell–Larcker criterion were examined. As shown in Table 3, all HTMT values were below the conservative threshold of 0.85, thereby confirming that each construct is empirically distinct from the others [85,86]. In addition, the Fornell–Larcker analysis indicated that the square root of each construct’s AVE exceeded its correlations with all other constructs, further validating discriminant validity. These results collectively affirm that the measurement model demonstrates adequate discriminant validity and conceptual distinctiveness across all constructs.

Second, discriminant validity was verified using the Fornell–Larcker criterion. In this analysis, the square root of the AVE for each construct was compared with the inter-construct correlation coefficients. As shown in Table 4, the square root of each construct’s AVE exceeded its correlations with all other constructs, thereby satisfying the Fornell–Larcker criterion [82]. These results provide additional evidence supporting the discriminant validity of the measurement model and confirm that each latent construct captures a unique conceptual dimension.

The analysis confirmed that the square root of the AVE for each construct was greater than its correlations with all other constructs, satisfying the Fornell–Larcker criterion [82]. Accordingly, these findings affirm that the measurement scales exhibit adequate discriminant validity, indicating that each construct captures a distinct theoretical dimension within the model.

### 5.2. Structural Model Assessment

Common method bias was assessed prior to hypothesis testing. Given that the data were collected using a single self-report survey, we assessed potential common method bias. Participation was voluntary and anonymous, and respondents were informed that there were no right or wrong answers. We examined common method bias using a full collinearity assessment approach. All VIF values were below the recommended threshold of 3.3, suggesting that common method bias was unlikely to confound the results [87]. To evaluate the structural model, several diagnostic criteria were examined, including the inner VIF, R^2^, and f^2^. The inner VIF values for all constructs were below the threshold of 3, indicating the absence of multicollinearity among the predictor variables [88]. Detailed results are presented in Table 5. Subsequently, R^2^ was used to assess the proportion of variance in the endogenous variables explained by their respective predictors. The model accounted for 71% of the variance in consumer inspiration, 53% in food pleasure, 50% in repurchase intention, 52% in WOM, and 48% in WPM (See Figure 2). These R^2^ values exceeded the 0.20 benchmark typically considered acceptable in consumer behavior research [84,85], demonstrating the substantial explanatory power of the proposed model. Subsequently, f^2^ values were examined to determine the individual contribution of exogenous constructs to the model. In accordance with Cohen’s [89] guidelines, f^2^ values between 0.02 and 0.15 indicate a small effect, 0.15–0.35 a medium effect, and values above 0.35 a large effect. The results revealed that most relationships exhibited moderate effect sizes (Table 5).

To address the potential generational bias, we added age as a control variable in the structural model (a single-item construct). The key path coefficients and their significance remained substantively unchanged, suggesting that our main findings are robust to the age composition of the sample.

After confirming both the measurement and structural model adequacy, the hypothesized relationships were tested using structural equation modeling (SEM) via SmartPLS 4. The results of the hypothesis testing, including path coefficients, t-values, and significance levels, are summarized in Table 5.

SEM analysis revealed that customization, trendiness, and advertising exerted significant positive effects on consumer inspiration, supporting H1c, H1d, and H1e. In contrast, entertainment and interaction did not demonstrate significant effects on consumer inspiration; thus, H1a and H1b were not supported. Regarding the nonsignificant paths from entertainment and interaction to consumer inspiration, collinearity diagnostics indicated no multicollinearity concerns (inner VIF values = 1.682 and 1.376, respectively). In addition, the effect sizes were negligible (f^2^ = 0.006 for entertainment; f^2^ = 0.004 for interaction), suggesting a very limited incremental contribution of these dimensions to explaining consumer inspiration. Furthermore, consumer inspiration exhibited a significant positive influence on repurchase intention, WOM recommendation, WPM, and food pleasure. Accordingly, H2a, H2b, H2c, and H3 were supported. In addition, food pleasure was found to have a significant positive effect on WOM recommendation and WPM, confirming H4b and H4c. However, the effect of food pleasure on repurchase intention was not statistically significant, leading to the rejection of H4a.

**Figure 3:**
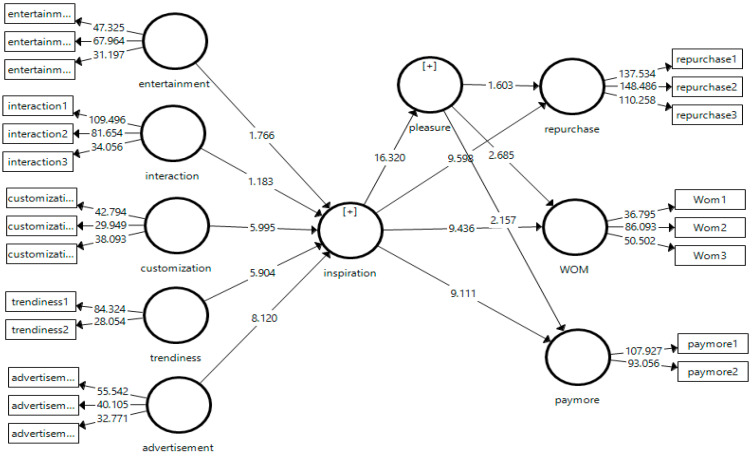
Hypothesis testing.

The corresponding path coefficients (β), significance levels (*p*-values), and R^2^ for these relationships are summarized in Figure 3 and Table 5. Collectively, these findings indicate that SMMAs dimensions—particularly customization, trendiness, and advertising—play a crucial role in fostering consumer inspiration, which subsequently enhances emotional responses (food pleasure) and behavioral intentions (repurchase, WOM, and WPM).

Finally, the mediating role of food pleasure in the relationship between consumer inspiration and behavioral intentions was examined using bootstrapping procedures in SmartPLS 4. The mediation analysis revealed that food pleasure significantly mediated the relationships between consumer inspiration and WOM recommendation and WPM, supporting H6 and H7 (Table 6).

However, the indirect effect of food pleasure on the relationship between consumer inspiration and repurchase intention was not statistically significant, indicating the absence of mediation. Consequently, H5 is not supported. These results, presented in Table 6, highlight that emotional gratification derived from food pleasure partially translates consumer inspiration into socially expressive (WOM) and value-based (WPM) behavioral outcomes, rather than habitual purchase behavior.

Since both the direct and indirect effects were statistically significant, the mediation pattern was identified as partial mediation, following the criteria proposed by Zhao et al. [90]. This indicates that food pleasure partially transmits the influence of consumer inspiration on behavioral intentions, whereas a portion of the effect remains direct.

## 6. Discussion

This study examined how SMMAs influence consumer inspiration and, indirectly, food pleasure and behavioral intentions in a hedonic food consumption context. Data from 425 Turkish participants who had experienced Dubai chocolate were analyzed using PLS-SEM. The results indicate that customization, trendiness, and advertising are the most influential SMMA dimensions in enhancing consumer inspiration (supporting H1c, H1d, and H1e). These relationships align with the view that marketing stimuli are more likely to elicit inspiration when they convey novelty and personal relevance [6,7]. Customization can enhance intrinsic motivation by providing tailored and personally relevant cues [64,65]. Trendiness can stimulate curiosity and discovery through perceptions of contemporaneity and social approval [4,30]. Advertising, especially when visually rich and story-driven, can support the inspirational process by creating vivid mental imagery [1]. Overall, the findings are consistent with prior studies reporting that SMMAs can stimulate consumer inspiration [2,5,91].

In contrast, entertainment and interaction did not provide significant incremental explanatory power for consumer inspiration in this setting (H1a and H1b not supported). Although these dimensions are often associated with engagement and emotional attachment [57,92], entertainment-oriented content may not always communicate novelty or personal meaning, resulting in temporary attention rather than inspiration [25]. Likewise, many platform interactions (for example, brief likes or comments) may lack the cognitive depth needed to trigger inspiration. In the case of a hedonic food product, such as a luxury chocolate brand, originality, trend value, and impactful promotional visuals may therefore be more inspiration-relevant than entertainment alone [8,9].

Consumer inspiration was positively associated with repurchase intention, WOM intention, WPM, and food pleasure (supporting H2a, H2b, H2c, and H3). In a motivational state, inspiration stimulates curiosity and exploration [6,7], which can strengthen consumers’ connection to the product and intensify the pleasure derived from consumption. These results are consistent with the SOR and PAD frameworks, which posit that external stimuli can shape organismic states and, in turn, behavioral responses [13]. The direct links between inspiration and multiple intentions further suggest that inspiration functions as a behavioral motivator rather than a purely affective reaction [7,29,93,94].

Food pleasure was positively related to WOM and WPM (supporting H4b and H4c), consistent with evidence that pleasurable experiences encourage social sharing and increase price tolerance [9,73]. The mediation analysis further showed that food pleasure partially mediates the relationships between inspiration and WOM and WPM (supporting H6 and H7), indicating an affective pathway through which inspiration translates into these outcomes [74,78]. However, food pleasure did not predict repurchase intention (H4a not supported) and did not mediate the relationship between inspiration and repurchase (H5 not supported). This pattern suggests that, in this context, repurchase intention may depend on mechanisms extending beyond immediate enjoyment. Accordingly, food pleasure appears more closely tied to experience-oriented outcomes (for example, sharing-related intentions) than to routine repeat-purchase intentions. Importantly, we do not claim that the current data identify the specific drivers of repurchase. Rather, prior literature suggests that repurchase can be shaped by stable or situational factors (for example, habit formation, price considerations, availability/accessibility, and trust), which were not measured here and should be examined in future research [36,92]. For hedonic and occasionally consumed products such as premium chocolate, consumers may also frame purchases as episodic indulgences, which could attenuate the direct role of pleasure in repurchase decisions.

### 6.1. Theoretical Implications

This study advances research on social media marketing and food consumer behavior by clarifying the roles of SMMAs, consumer inspiration, and food pleasure in explaining behavioral intentions. First, it contributes to the emerging stream that integrates consumer inspiration into SMMA research by showing that inspiration is most strongly related to dimensions emphasizing personal relevance and novelty (customization, trendiness, and advertising) [6,7]. This finding supports the view that inspiration is a motivational state that can arise through meaning-making processes triggered by marketing stimuli.

Second, the lack of incremental effects for entertainment and interaction suggests an important boundary condition: not all SMMA dimensions are equally relevant for generating inspiration. This result is consistent with work emphasizing the importance of cognitive depth, novelty, and personal relevance for inspiration to occur [25,65], and it helps distinguish inspiration from broader engagement constructs in social media contexts.

Third, by combining hedonic consumption theory [12] with the SOR paradigm [13], this study clarifies a mechanism whereby inspiration relates to behavioral outcomes through hedonic pleasure. The mediating role of food pleasure in WOM and WPM aligns with evidence that emotional transfer mechanisms can connect motivational states to consumer actions [74,78]. Finally, the absence of a direct food pleasure → repurchase link offers a boundary condition for hedonic consumption research, suggesting that pleasurable experiences may not necessarily translate into continuous repurchase, particularly for episodic indulgence products [36,92].

### 6.2. Practical Implications

The findings offer actionable guidance for food and chocolate brands seeking to improve the effectiveness of digital marketing. First, the prominence of customization, trendiness, and advertising in stimulating consumer inspiration highlights the value of designing content that is personalized, trend-relevant, and visually compelling. Marketers may emphasize innovative flavor combinations, product storytelling, and aesthetically appealing presentations to evoke curiosity and discovery and enhance positive affect toward the brand.

Second, because entertainment and interaction did not add meaningful explanatory power for inspiration in this context, campaigns may benefit from moving beyond “fun” content toward formats that create personal relevance and stronger meaning. For example, interactive initiatives that invite consumers to share personal experiences (for example, branded hashtags) may be more effective when they are designed to elicit stories, discovery, and identity-relevant expression rather than brief, low-effort interactions.

Third, the links from inspiration to food pleasure and behavioral intentions indicate that inspiration-based strategies can strengthen advocacy-related outcomes and value perceptions. For hedonic products such as Dubai chocolate, visually rich, emotionally toned, and story-driven advertisements may be particularly effective in shaping WOM and WPM. Finally, because food pleasure alone did not translate into repurchase intention, brands aiming to support repeat purchases may need to complement emotional appeal by reinforcing cues related to consistent quality, accessibility, perceived fairness of price, and trust.

### 6.3. Limitations and Directions for Future Research

This study makes a meaningful contribution to the hedonic food consumption literature by examining the effects of SMMAs on consumer inspiration, food pleasure, and behavioral intentions. However, the findings should be interpreted considering several limitations. First, the sample in this study was limited to individuals residing in Türkiye who actively use social media and have experienced Dubai chocolate. This contextual specificity restricts the generalizability of the findings to other cultural environments or brand types. Future research could address this limitation by comparing consumer samples across different countries to examine how cultural differences influence consumer inspiration and hedonic consumption patterns. Second, this study focused exclusively on a hedonic food product—a luxury chocolate brand. Therefore, the results cannot be directly generalized to functional or utilitarian product categories. Future studies may compare different product types, such as healthy snacks, coffee brands, or gastronomic experiences, to explore how inspiration operates differently across hedonic and utilitarian consumption contexts. Third, the nonsignificant effect of food pleasure on repurchase intention suggests that emotional variables may have limited influence on long-term behavioral outcomes. This finding offers an opportunity for future research to integrate additional constructs, such as satisfaction, trust, habit, and price perception, into extended models. Employing multilevel mediation or longitudinal designs could further elucidate the dynamic relationships between inspiration, emotional experience, and consumer loyalty over time. Fourth, although the sample included multiple age brackets, it was skewed toward younger respondents (approximately 69% were under 35 years). This may limit the generalizability of the findings to older cohorts, as social media usage intensity and hedonic consumption motivations may differ by age/generation. Therefore, the results should be interpreted primarily as reflecting younger consumers, and future research should employ more age-balanced sampling strategies and cross-cohort validations. Finally, although this study captured perceived food pleasure through self-report measures in a naturalistic setting, future research could strengthen methodological rigor by incorporating sensory methods (for example, consumer hedonic tests, sensory profiling) and experimental designs (for example, controlled exposure to specific social media stimuli followed by tasting tasks). Such approaches would allow a more direct assessment of sensory-driven pleasure and provide stronger evidence regarding causal mechanisms.

## 7. Conclusions

This study tested a model linking SMMAs to consumer inspiration and subsequently to food pleasure and behavioral intentions in the context of Dubai Chocolate. The results show that customization, trendiness, and advertising are the strongest SMMA drivers of consumer inspiration, whereas entertainment and interaction do not exhibit significant incremental effects. Consumer inspiration is positively associated with food pleasure and behavioral intentions, indicating that inspiration is a key motivational state in this hedonic social-media-driven consumption setting.

Food pleasure partially transmits the effect of inspiration on WOM intention and WPM but does not directly predict repurchase intention. Overall, the findings suggest that inspiration-driven enjoyment is more closely tied to experience-oriented outcomes (for example, advocacy and value-related intentions) than to routine repeat-purchase intentions for this product. A detailed discussion of the theoretical and managerial implications, as well as the limitations and future research directions, is provided in Section 6.1, Section 6.2 and Section 6.3.

## Figures and Tables

**Table 1:** Demographic characteristics of the participants.

Demographic Characteristics		*n*	%
Age	˂18	64	15.1
	18–24	126	29.6
	25–34	102	24.0
	35–44	67	15.8
	45–54	35	8.2
	55–64	18	4.2
	≥65	13	3.1
Gender	Female	206	48.5
	Male	219	51.5
Marital status	Married	184	43.3
	Single	241	56.7
Education	Primary education	13	3.1
	High School	38	8.9
	Associate degree	126	29.6
	Bachelor’s degree	187	44.0
	Master’s degree/Ph.D.	61	14.4
Self-reported (perceived) income level	Very low	62	14.6
	Low	106	24.9
	Average	172	40.5
	High	58	13.6
	Very high	27	6.4
The most frequently used social media platform	Instagram	116	27.3
	Facebook	63	14.8
	X	51	12.0
	YouTube	66	15.5
	TikTok	81	19.1
	Snapchat	38	8.9
	Other	10	2.4

**Table 2:** Reliability and validity (overall sample).

Items	Factor Loading
Entertainment	
α = 0.877; CR = 0.924; AVE = 0.802	
This product (Dubai chocolate) is enjoyable on social media.	0.904
The content shared by this product on social media is enjoyable.	0.915
Social media posts on these products are interesting.	0.868
Interaction	
α = 0.870; CR = 0.921; AVE = 0.795	
Information regarding this product is shared on social media.	0.926
Discussions and exchanges of ideas about this product occur on social media pages.	0.910
Expressing opinions about this product is easy on social media.	0.836
Customization	
α = 0.743; CR = 0.853; AVE = 0.660	
I can find the information I need about this product in its social media accounts.	0.839
Social media provided me with the information I needed about this product.	0.767
Thanks to the guidance on this product’s social media accounts, I can easily access the information I need.	0.829
Trendiness	
α = 0.705; CR = 0.870; AVE = 0.770	
The social media posts of this product are up to date.	0.907
The social media usage of this product is fashionable.	0.846
Advertisement	
α = 0.755; CR = 0.859; AVE = 0.671	
I like the product ads that are posted on social media.	0.846
The advertisements for this product on social media are interesting.	0.806
This product’s social media ads positively influenced my interest in the product.	0.804
Consumer Inspiration (CI)	
α = 0.929; CR = 0.940; AVE = 0.612	
Social media tools have sparked my imagination.	0.832
Thank you for bringing this new idea to our attention through social media tools.	0.811
By exploring social media tools, I unexpectedly and spontaneously gained new ideas.	0.816
I am amazed by the new ideas shared on social media tools.	0.780
Social media tools have helped me discover something new.	0.748
Social media tools have inspired me to do something.	0.789
Social media tools sparked my desire to do something.	0.784
Social media tools have increased my interest in doing something.	0.778
Social media tools motivated me to do something.	0.763
Social media tools have sparked in me a desire to do more.	0.717
Food Pleasure (FP)	
α = 0.915; CR = 0.930; AVE = 0.596	
The emergence of Dubai chocolate made me happy.	0.724
The smell of Dubai chocolate makes me feel very happy.	0.767
The taste of Dubai chocolate makes me feel very happy.	0.807
Eating Dubai chocolate reminds me of the flavors of my childhood.	0.726
The taste of Dubai chocolate reminds me of a familiar taste.	0.802
Dubai chocolate is a completely natural food, as expected.	0.788
Dubai chocolate product information makes me happy.	0.800
The environmentally friendly production process of Dubai chocolate makes me happy.	0.798
Dubai chocolate brings me spiritual happiness.	0.727
Repurchase Intention (RI)	
α = 0.951; CR = 0.968; AVE = 0.911	
I plan to buy the Dubai chocolate I have seen on social media.	0.956
I plan to buy Dubai chocolate that I like based on social media interactions.	0.959
I am very likely to buy the Dubai chocolate recommended by my friends on social media.	0.948
WOM Intention	
α = 0.866; CR = 0.918; AVE = 0.789	
I will post positive reviews about Dubai chocolate on social media.	0.855
I will recommend Dubai chocolate through social media.	0.916
I will recommend Dubai chocolate to my social media acquaintances.	0.893
WPM	
α = 0.867; CR = 0.938; AVE = 0.882	
I will pay more for Dubai chocolate than for similar products.	0.940
I intend to buy Dubai chocolate even if another brand advertises at a lower price.	0.939

**Table 3:** Discriminant validity analysis results (HTMT).

Variables	1	2	3	4	5	6	7	8	9	10
Entertainment										
Interaction	0.429									
Customization	0.592	0.555								
Trendiness	0.675	0.535	0.792							
Advertisement	0.726	0.595	0.816	0.801						
CI	0.532	0.450	0.811	0.777	0.818					
FP	0.468	0.428	0.715	0.659	0.777	0.790				
RI	0.311	0.404	0.802	0.511	0.716	0.749	0.600			
WOM	0.318	0.462	0.812	0.557	0.838	0.793	0.669	0.801		
WPM	0.326	0.417	0.797	0.529	0.788	0.770	0.635	0.826	0.814	

**Table 4:** Discriminant validity analysis results (Fornell Larcker Criterion).

Variables	1	2	3	4	5	6	7	8	9	10
Entertainment	**0.896**									
Interaction	0.374	**0.891**								
Customization	0.482	0.445	**0.812**							
Trendiness	0.520	0.421	0.574	**0.877**						
Advertisement	0.597	0.476	0.715	0.580	**0.819**					
CI	0.482	0.404	0.765	0.636	0.774	**0.782**				
FP	0.419	0.383	0.589	0.534	0.639	0.729	**0.772**			
RI	0.284	0.368	0.762	0.426	0.610	0.707	0.563	**0.954**		
WOM	0.279	0.402	0.718	0.444	0.694	0.713	0.597	0.877	**0.888**	
WPM	0.285	0.363	0.722	0.419	0.639	0.693	0.568	0.918	0.884	**0.939**

**Note**: Bold font represents the AVE square root value.

**Table 5:** Structural properties.

Hypotheses		β	T Statistics	*p* Values	InnerVIF	f^2^
H_1a_	Entertainment » CI	−0.055	1.766	0.077	1.682	0.006
H_1b_	Interaction » CI	−0.037	1.183	0.237	1.376	0.004
H_1c_	Customization » CI	0.376	5.995	0.000 ***	2.256	0.222
H_1d_	Trendiness » CI	0.216	5.904	0.000 ***	1.780	0.090
H_1e_	Advertisement » CI	0.429	8.120	0.000 ***	2.620	0.249
H_2a_	CI » RI	0.634	9.598	0.000 ***	2.134	0.379
H_2b_	CI » WOM	0.594	9.436	0.000 ***	2.134	0.345
H_2c_	CI » WPM	0.595	9.111	0.000 ***	2.134	0.323
H_3_	CI » FP	0.730	16.320	0.000 ***	1.000	1.134
H_4a_	FP » RI	0.100	1.603	0.109	2.134	0.010
H_4b_	FP » WOM	0.164	2.685	0.007 **	2.134	0.027
H_4c_	FP » WPM	0.134	2.157	0.031 *	2.134	0.017

*p* = < 0.001 ***, *p* = < 0.01 **, *p* = < 0.05 *.

**Table 6:** Mediation effect analysis results.

Hypotheses		β	T Statistics	*p* Values
H5	CI » FP » RI	0.073	1.540	0.124
H6	CI » FP » WOM	0.119	2.520	0.012 *
H7	CI » FP » WPM	0.098	2.046	0.041 *

*p* = < 0.05 *.

## Data Availability

The data analyzed in this study are available upon reasonable request from the corresponding author.

## Abbreviations

The following abbreviations are used in this manuscript:

SMMAs	Social Media Marketing Activities
PLS-SEM	Partial Least Squares Structural Equation Modeling
SOR	Stimulus–Organism–Response
PAD	Pleasure–Arousal–Dominance
WOM	word-of-mouth
PLSc	Partial Least Squares-consistent
